# Survival of Peritoneal Membrane Function on Biocompatible Dialysis Solutions in a Peritoneal Dialysis Cohort Assessed by a Novel Test

**DOI:** 10.3390/jcm10163650

**Published:** 2021-08-18

**Authors:** Olga Balafa, Anila Duni, Paraskevi Tseke, Karolos Rapsomanikis, Paraskevi Pavlakou, Margarita Ikonomou, Vasileios Tatsis, Evangelia Dounousi

**Affiliations:** 1 Department of Nephrology, University Hospital of Ioannina, 45500 Ioannina, Greece; anikristduni@yahoo.com (A.D.); karolospaulos@hotmail.com (K.R.); pavlakoup@gmail.com (P.P.); ritaikonomu@yahoo.gr (M.I.); evangeldou@gmail.com (E.D.); 2 Department of Nephrology, General Hospital Alexandra, 11528 Athens, Greece; tsekedaki@hotmail.com; 3 Department of Surgery, University Hospital of Ioannina, 45500 Ioannina, Greece; vtatsis@gmail.com

**Keywords:** longitudinal, dialysis, biocompatible, technical survival

## Abstract

Background: Longitudinal surveillance of peritoneal membrane function is crucial in defining patients with a risk of ultrafiltration failure. Long PD is associated with increased low molecular weight solute transport and decreased ultrafiltration and free water transport. Classic PET test only provides information about low molecular solute transport, and the vast majority of longitudinal studies are based on this test and include patients using conventional dialysates. Our aim was to prospectively analyze longitudinal data on peritoneal function in patients on biocompatible solutions using a novel test. Methods: Membrane function data were collected based on uni-PET (a combination of modified and mini PET). A total of 85 patients (age 61.1 ± 15.1 years) with at least one test/year were included. Results: The median follow up was 36 months (21.3, 67.2). A total of 219 PETs were performed. One-way repeated measures ANOVA showed that there were no statistically significant differences over time in ultrafiltration, free water transport, ultrafiltration through small pores, sodium removal, D/D0 and D/PCre in repeated PET-tests. Twenty-three tests revealed ultrafiltration failure in 16 (18.8%) patients. Those patients were longer on PD, had higher D/P creatinine ratios, lower ultrafiltration at one hour with lower free water transport and higher urine volume at baseline. Multivariate analysis revealed that the variation of ultrafiltration over repeated PET-tests independently correlated only with D/Pcreatinine, free water transport and ultrafiltration through small pores. Conclusions. Uni-PET is a combination of two tests that provides more information on the function of the membrane compared with PET. Our study on a PD cohort using only biocompatible solutions revealed that function membrane parameters remained stable over a long time. Ultrafiltration failure was correlated with increased D/P creatinine and decreased free water transport and ultrafiltration through small pores.

## 1. Introduction

Ultrafiltration (UF) failure is an important complication of peritoneal dialysis (PD) in long-term patients, and it causes reduced patient and technique survival [1,2]. UFF is defined by the International Society for PD [3] as: net UF less than 400 mL after drainage of a 4% (3.86 or 4.25%, depending on the manufacturer) dialysis solution with an intraperitoneal stay of 4 h. The majority of longitudinal studies show a gradual increase in low molecular weight solute transport in the years following PD initiation and a decrease of net ultrafiltration and small-pore fluid transport, especially after 4 years of PD [4]. Ultrafiltration failure is not only associated with fast small solute transport rates but also with decreased free water transport (FWT) and osmotic conductance [4]. Encapsulating peritonitis is the most devastating complication of long PD, and it is characterized by an early and significant reduction of FWT [5]. Therefore, longitudinal surveillance of peritoneal membrane function in PD patients is crucial, as it can detect changes and guide prescription and clinical decisions, such as timely transfer to hemodialysis.

The peritoneal equilibration test (PET) [6] is the most popular test for evaluating the function of the peritoneal membrane, but it only provides data about the kinetics of the small molecular weight solutes. Different tests have been developed, such as a modified PET with 3.86% glucose dialysate, a mini PET test and a PDC test that can give more information about FWT, osmotic conductance, sodium sieving and UF failure [7]. Recent ISPD guidelines encourage the use of these tests [8], but unfortunately, none of them can give all the relevant information concurrently. La Milia [9] and Cnossen [10] et al. described a combination of modified and mini PET test in order to benefit from the advantages of both tests (called uni-PET or two-in-one modified PET).

However, unlike the classic PET for which longitudinal data are widely available, sparse data have so far been collected with two-in-one modified PET [11,12]. In addition, few studies are conducted with patients using solely biocompatible solutions. The aims of our study were to: (a) analyze the time course of prospectively collected observational data on peritoneal function in consecutive PD patients using two-in-one modified PET and biocompatible solutions (b) evaluate UF failure and association with clinical and membrane characteristics.

## 2. Materials and Methods

We enrolled patients from our PD Unit from January 2013 till December 2019 who underwent at least one test within 12 months of the start of PD and then once a year. Written informed consent was given by all patients included in the study. Τhe study was approved by the Ethics Committee of our hospital. At the time of the test, all the patients had been peritonitis-free for at least 4 weeks. The test was performed on an annual basis as peritoneal membrane routine evaluation. All patients used biocompatible PD solutions with low GDPs-neutral ph (Balance, Fresenius©, Bad Homburg, Germany or Physioneal, Baxter©, Baxter Healthcare Ltd., IRL-Dublin, Ireland) according to the patient‘s prescription. In our unit, biocompatible dialysates are used exclusively since 2002 due to reimbursement reasons. Both automated PD (APD) and continuous ambulatory PD patients were included. The use of one aminoacid–based solution or one icodextrin exchange was allowed.

The test was performed using a 3.86% glucose dialysate and temporal drainage at one hour and final drainage at 4 h. In that way, the test gives information for small solutes kinetics (D/P creatinine), sodium sieving, free water transport, ultrafiltration and ultrafiltration failure. Overfill of the bags was taken into consideration; the volume of the fresh dialysate and the drained dialysate was measured by weighing the bag and then subtracting the weight of the empty bag. At one hour, the cavity was emptied, the ultrafiltration was measured, a sample was taken, and the dialysate was re-instilled. At 240 min, the whole drained volume was measured, after collecting by gravity for at least 20 min. In total, a blood sample was taken at 60 min, while 10 mL dialysate samples were taken at 0, 60 and 240 min. The patients were instructed to sit up or move in bed before drawing each dialysate sample. Data collection also included baseline demographics, primary kidney disease, co-morbidities and routine laboratory exams.

### 2.1. Calculations

1 We estimated classic peritoneal transport parameters such as D/P creatinine, D/P urea,

D/D0(glucose) and ultrafiltration (UF) at 4 h and at 60 min

2 We calculated dipping of sodium expressed as

D0Na-D1Na or Dip/DPNa = (Dialysate sodium time 0/plasma sodium) − (Dialysate sodium time 1 h/plasma sodium)

3 Free water transport (FWT) was calculated as follows:

FWT(mL) = Total UF volume at 60 min(mL) − UF through the small pores at 60 min (mL)

where the UF through the small pores (UFSP) is calculated as follows:

UFSP (mL) = [sodium removal (mmol) × 1000]/Psodium

4 Sodium removal = [Dialysate V in 1 h (L) × Dialysate sodium at 1 h (mmol/L)] − [Dialysate V instilled (L) × Dialysate sodium at 0 min (mmol/L)]

Correction of FWT by Venturoli and Rippe was applied as follows [13]:

FWT corrected = Total UF at 1 h + 15 − 0.92 × UFSP

### 2.2. Statistical Analysis

Data are presented as mean ± sd (normally distributed continuous data), median, IQR (interquantile range, not-normally distributed continuous data) or percentage (discrete data). In the comparison between patients, *p*-values are estimated with t-test and Mann–Whitney test. One-way repeated-measures ANOVA was run to determine differences in variables with repeated observations over time. Univariate random effects logistic time-series models and multivariate generalized estimating equations for analyzing time-series data with robust standard errors were applied to determine if there was any association structure between repeated measurements. In multivariate analysis, all statistically significant variables (that were identified in univariate analysis, *p* = 0.1) were included, and by using the stepwise procedure, all variables that showed collinearity were excluded. Analysis was performed using Stata 14.0 (Copyright 1985–2019; StataCorp LP, College Station, TX, USA).

## 3. Results

We included 85 patients (male to female 51/34, age 61.1 ± 15.1 years). The median follow up was 36 months (IQR 21.3, 67.2). Primary renal diseases were glomerulonephritis in 24 patients (28.2%), ischaemic/hypertensive nephropathy in 20 (23.5%), diabetic nephropathy in 13 (15.3%), polycystic disease in 5 (5.8%) and unknown in 23 patients (27%). The median time to the first PET was 113.5 days (Table 1), while the median PD vintage was 35.6 months. Baseline peritoneal transport characteristics are shown in Table 2. Ultrafiltration UF (at 4 h) was 655 ± 265 mL, D/P creatinine 0.74 ± 0.11 and D/D0 glucose 0.29 (IQR 0.24, 0.33). At one hour, Dip DPNa was 0.05 ± 0.04, FWT 175.20 ± 50.40 mL, FWT (corrected) 214.9 ± 194.7 mL while UF60 was 375 mL (IQR 250, 480) and UFSP 235.6 mL (IQR 111.3, 315.4)

Patients were then evaluated longitudinally. During the study period, 235 uni-PETs were performed, and 16 tests were excluded due to violations of the test protocol (inaccurate time, one-way obstruction, dialysate spill) (Figure S1).

**Figure 1:**
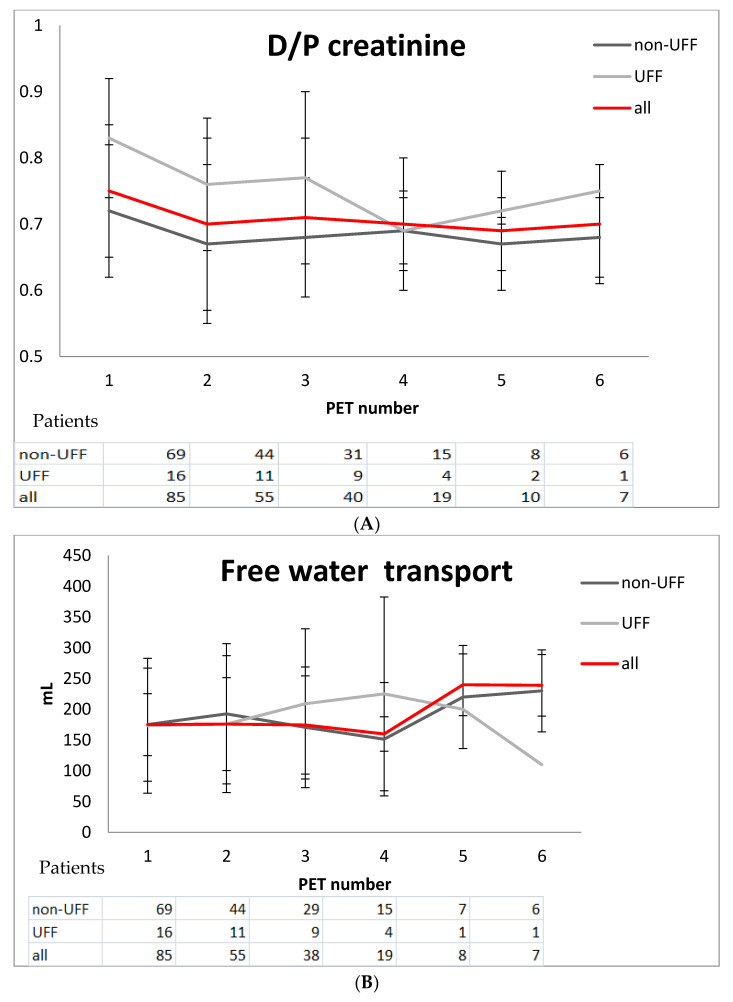

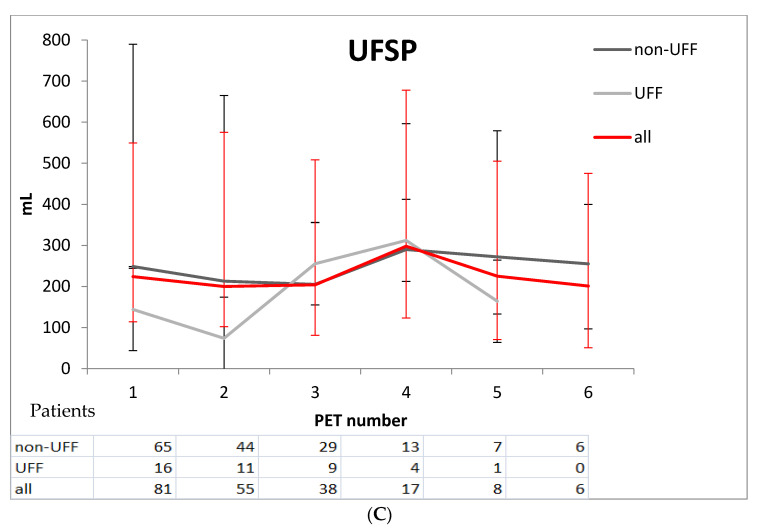
Changes of membrane characteristics over time in patients with UFF and non-UFF; (**A**) D/Pcre, (**B**) Free Water and Transport (**C**). Ultrafiltration through small pores (UFSP).

Finally, a total of 219 uni-PETs were included in the analysis. Five and six tests were available in 10 and 7 patients, respectively. There were no statistically significant correlations between baseline weight, age, albumin levels, gender or duration onPD with ultrafiltration at any time point. One-way repeated measures ANOVA in all patients showed that there were no statistically significant differences over time in UF (*p* = 0.79), FWT (*p* = 0.45), UFSP (*p* = 0.37), Na removal (*p* = 0.36), D/D0 (*p* = 0.25) and D/PCre (*p* = 0.23) in repeated uni-PET tests. The observed values of D/Pcre, FWT, UFSP (Figure 1), Dip/DPNa and UF remained rather stable across the different time measurements.

During the follow-up period, a total of 23 uni-PET tests revealed ultrafiltration failure. UFF was noted in 16 (18.8%) patients, whose characteristics are shown in Table 3. These patients were transferred to hemodialysis. Patients with UFF were longer on PD, had higher D/P creatinine ratios, lower ultrafiltration at one hour with lower FWT and higher urine volume at baseline. In order to identify predictors of UFF, we performed further univariate analysis random effects logistic time-series models. Age at start, sex, TPR, albumin, creatinine, urea, kt/vperitoneal, kt/vrenal and time in PD (when each test was performed) were not significantly associated with UFF development. Conversely, DD0 (OR = 0.84, *p* = 0.001), urine volume (OR = 1.26, *p* = 0.027 (per 100 mL increase), D/Pcre (OR = 1.15, *p* < 0.001 (per 0.01 increase)), Na removal (OR = 0.99, *p* = 0.003), UFSP (OR = 0.98, *p* = 0.003) were significantly associated with relative risk of UFF development. In multivariate analysis (generalized estimating equations for analyzing time-series data with robust standard errors) the variation of UF over repeated tests independently correlated with D/P cre, UFSP and FWT, as shown in Table 4. Membrane characteristics such as D/Pcre, FWT and UFSP showed changes over time in UFF patients compared with non-UFF (Figure 1). These changes were evident in a median time of 36.9 months after PD start.

## 4. Discussion

Our prospective study on peritoneal membrane function in a PD cohort using only biocompatible solutions reveals that function parameters remain rather stable over a long time, despite the common belief that solute and water transport changes. We collected this information based on a simple and modified PET (uni-PET), whose relevant publications are limited. A minority of patients (18.8%) developed ultrafiltration failure and were transferred to hemodialysis, which was correlated with increased D/P cre and decreased UFSP and FWT.

According to three-pore theory, small pores of the peritoneal membrane allow the transport of water (small pore transport-SPT) and low molecular weight molecules, while aquaporins (water channels) are responsible for the transport of water only, so called free water transport. These water channels explain the sodium sieving phenomenon too, which is a decrease in sodium in the dialysate in one hour of hypertonic solution exchange [14,15]. Long-term ultrafiltration failure is associated with fast small solute transport rates, decreased FWT, decreased SPT and osmotic conductance (represents the ability of glucose to exert an osmotic pressure sufficient to cause transperitoneal ultrafiltration) [16]. The increased small solute transport rates are likely caused by an increase in the number of peritoneal vessels over time that becomes evident after 2 years or more, as morphological studies have demonstrated [17]. Very low values for FWT are present in patients with encapsulating peritoneal sclerosis [5].

The original PET test is the simplest, commonly used test for the evaluation of peritoneal membrane function. It uses a fixed fill volume of 2 L of a 2.27% glucose solution over a 4-h dwell and provides an estimation of small-solute transport, as expressed by D/Pcreatinine and D/D0 for glucose. La Milia proposed the mini test (3.86% solution and 1 h dwell) for studying FWT and sodium sieving, but the test cannot estimate solute transport rates and UF at four hours as classic PET does [9]. The combination of mini and 4-h PET with a 3.86% solution is a clever way of incorporating info about fluid and solute transport in detail. The use of a 3.86% glucose solution not only provides larger drained volumes and diminishes the probability of measurement mistakes but can define UFF at 4 h, as ISPD guidelines propose. In our study, the use of uni-PET (two-in-one modified) has been proven simple, easy to be performed by the nursing staff, and it has provided extended information for membrane function of our patients, cross-sectionally and longitudinally. To our knowledge, only one relevant longitudinal study (based on uni-PET) has been published, but all of the patients included were only treated with conventional solutions [11].

The majority of studies on peritoneal membrane changes over time are mainly based on classic PET data [18,19]. The longitudinal study by Davies of patients treated with convention solutions reported that late increases in small solute transport were associated with a fall in UF capacity, especially in those individuals with an increased dialysate glucose exposure, suggesting a causal relationship between glucose and peritoneal morphological changes. Peritonitis episodes seem to influence the time-course of small-solute and fluid transport—especially FWT in some studies [20], while other studies failed to prove a correlation between peritonitis and membrane function changes [21,22].

In our study, stability of membrane characteristics over time (D/P ratios, FWT, sodium removal, UF) could be attributed to the absolute use of biocompatible dialysates and sparse use of hypertonic solutions. Moreover, peritonitis incidence in our unit is extremely low in the study period (from 0.01 to 0.16 episodes per patient years), which could also contribute to the steady membrane characteristics over time. Few studies have addressed the longitudinal membrane changes of patients using biocompatible solutions comparing with conventional ones. The so-called biocompatible dialysates contain a reduced amount of glucose degradation products and neutral pH, but glucose still remains the main osmotic factor in the same concentrations as in conventional solutions. The Balance in Australia and New Zealand Peritoneal Dialysis Patients (BalANZ) Study found various changes of peritoneal solute transport rates (PSTR) over time in both solutions; biocompatible ones seemed more beneficial; however, the study only lasted two years [23]. A multicenter, observational, long-term study [19] compared solute transport rates in biocompatible vs. standard solutions and found that D/P creatinine increased between 3.5 and 7 years of treatment in standard solutions, while with biocompatible ones, the rate was lower at baseline, with a steeper initial rise in PSTR. By 2 years of therapy, PSTR was similar for both solutions and no further increase in PSTR in patients using biocompatible solutions between years 2 and 4 of treatment. Van Diepen et al. [24], in a similar study based on SPA test data, found that conventional fluids showed an increase in small solute transport and a decrease of free water transport, while changes were minor or absent in the biocompatible treatment group. Regarding solute transport rates, we reconfirmed the plateau during a 6-year follow-up, as studies [23] and [24] did. Regarding total UF, SPT and FWT (at 60 min), our patients presented no significant changes. FWT and UFSP only decreased over time in patients who developed UFF, which could be attributed to peritoneal vascular changes due to the accumulation of glucose products [25]. These changes were evident about 3.5 years after PD start in our cohort.

La Milia et al. [11] used uni-PET like us to assess longitudinal data on membrane function in 95 patients and only found significant changes in total UF and sodium removal. Comparing with our study, all patients were treated with conventional dialysates, and the median follow-up was lesser than ours (25 vs. 36 months). Our results about the correlation of UFF with FWT and D/P creatinine agree with their results. However, LaMilia et al. provided no data about small pore transport (UFSP).

Our study has limitations: a mixed population of prevalent and incident patients from a single PD unit and absence of direct comparison with conventional dialysates. On the other hand, the fact that the data were collected from only one center provided strong and reproducible measurements based on a strictly standardized modified PET used for more than a decade in our unit. The strengths of our study are its prospective and longitudinal design, a fairly large number of patients included and extended information on solute and fluid transport based on the uni-PET test.

## 5. Conclusions

We proved that this test is a clever combination of two tests that could substitute the classical 2.27%-PET, as it allows better observation of the function of the peritoneal membrane. Peritoneal membrane characteristics remained stable over time in this PD cohort using biocompatible dialysates. Furthermore, we showed some interesting alterations of the function of the peritoneal membrane over time in patients developing UFF, such as the progressive decrease of the FWT and small pore transport, parameters that should be taken into account in the follow up of long-term patients. We should emphasize the fact that UFF correlated only with these specific membrane function characteristics and not clinical parameters, and this underlines the significance of longitudinal surveillance assessed by detailed tests, such as the uni-PET.

## Figures and Tables

**Table 1:** Baseline clinical data.

Age (years)	61.15 ± 15.12
Gender (male-female)	51(60%)–34(40%)
Weight (kgr)	72.61 ± 13.06
Time to 1st PET (days)	113.50 (IQR 58.50, 497.50)
Primary kidney disease	
glomerulonephritis	24 (28.2%)
ischaemic/hypertensive nephropathy	20(23.5%)
diabetic nephropathy	13(15.3%),
polycystic disease	5(5.8%)
unknown	23 (27%)
Co-morbidities	
Diabetes	20 (23.5%)
Coronary atheromatic disease	14 (16.5%)
Peripheral vascular disease	12(14.1%)
Congestive Heart failure	5 (5.8%)
APD	20 (23.5%)
Albumin (g/dL)	3.64 ± 0.38
Urea (mg/dL)	115 (IQR 105, 137)
Creatinine (mg/dL)	5.71 (IQR 4.62, 8.20)
KT/V peritoneal	1.02 (IQR 0.82, 1.27)
Residual GFR(mL/min/1.73 m^2^)	10.2 ± 4.12
Daily urine volume (mL)	1312.40 ± 700.20

Data are presented as mean ± sd (normally distributed continuous data), median, IQR (interquantile range, not-normally distributed continuous data) or percentage.

**Table 2:** Baseline peritoneal transport characteristics.

60 min	
DipDPNa	0.05 ± 0.04
UF60 (mL)	375 (IQR 250, 480)
UFSP (mL)	235.60 (IQR 111.30, 315.40)
FWT (mL)	175.20± 50.4
corrected FWT (mL)	214.90 ± 194.70
240 min	
D/P creatinine	0.74 ± 0.11
D/D0 glucose	0.29 (IQR 0.24, 0.33)
UF 240 (mL)	655 ± 265

Data are presented as mean ± sd (normally distributed continuous data), median, IQR (interquantile range, not-normally distributed continuous data) or percentage.

**Table 3:** A comparison between patients with non-ultrafiltration failure (UFF) vs. UFF.

	Non UFF (*n* = 69)	UFF(*n* = 16)	*p*-Value
Age (years)	64.80 (IQR 51.90, 72.50)	63.30 (IQR 49.60, 70.60)	0.6
Gender (male/female)	26/43	8/8	0.47
PD duration (months)	34.40 (IQR 21.30, 63.30)	49.90 (IQR 26.60, 83.60)	0.07
UF (mL)	722 (IQR 622, 848)	349 (IQR 120.5, 375)	<0.001
D/P cre	0.71 (IQR 0.66, 0.76)	0.83 (IQR 0.77, 0.89)	<0.001
Dip D/PNa	0.048 (IQR 0.03, 0.07)	0.04 (IQR 0.01, 0.08)	0.19
UF60 mL	391 (IQR 292, 480)	265 (IQR 1, 391)	<0.001
FWT mL	175.20 (IQR 126.70, 242.10)	173.30 (IQR 58.50, 202.20)	0.01
Urine volume (mL)	1272.10 ± 677.40	1490.80 ± 800.10	0.01

**Table 4:** Multivariate analysis of UF over repeated uni-PET tests *(Random effects logistic regression)*.

	Standardized *β* Coefficient	*z*-Test	*p*-Value
D/P cre (0.01 increase)	13.9	3.36	0.001
FWT (mL)	−0.0084	−2.44	0.015
UFSP (mL)	−0.0078	−2.59	0.01

## Supplementary Materials

The following are available online at https://www.mdpi.com/article/10.3390/jcm10163650/s1, Figure S1: Flowchart of patients and PET tests during the study.
