# Supplementary figures and images for: Survival of Peritoneal Membrane Function on Biocompatible Dialysis Solutions in a Peritoneal Dialysis Cohort Assessed by a Novel Test
Source: J Clin Med. 2021 Aug 18;10(16):3650. doi: 10.3390/jcm10163650 (PMC8396924; doi:10.3390/jcm10163650)

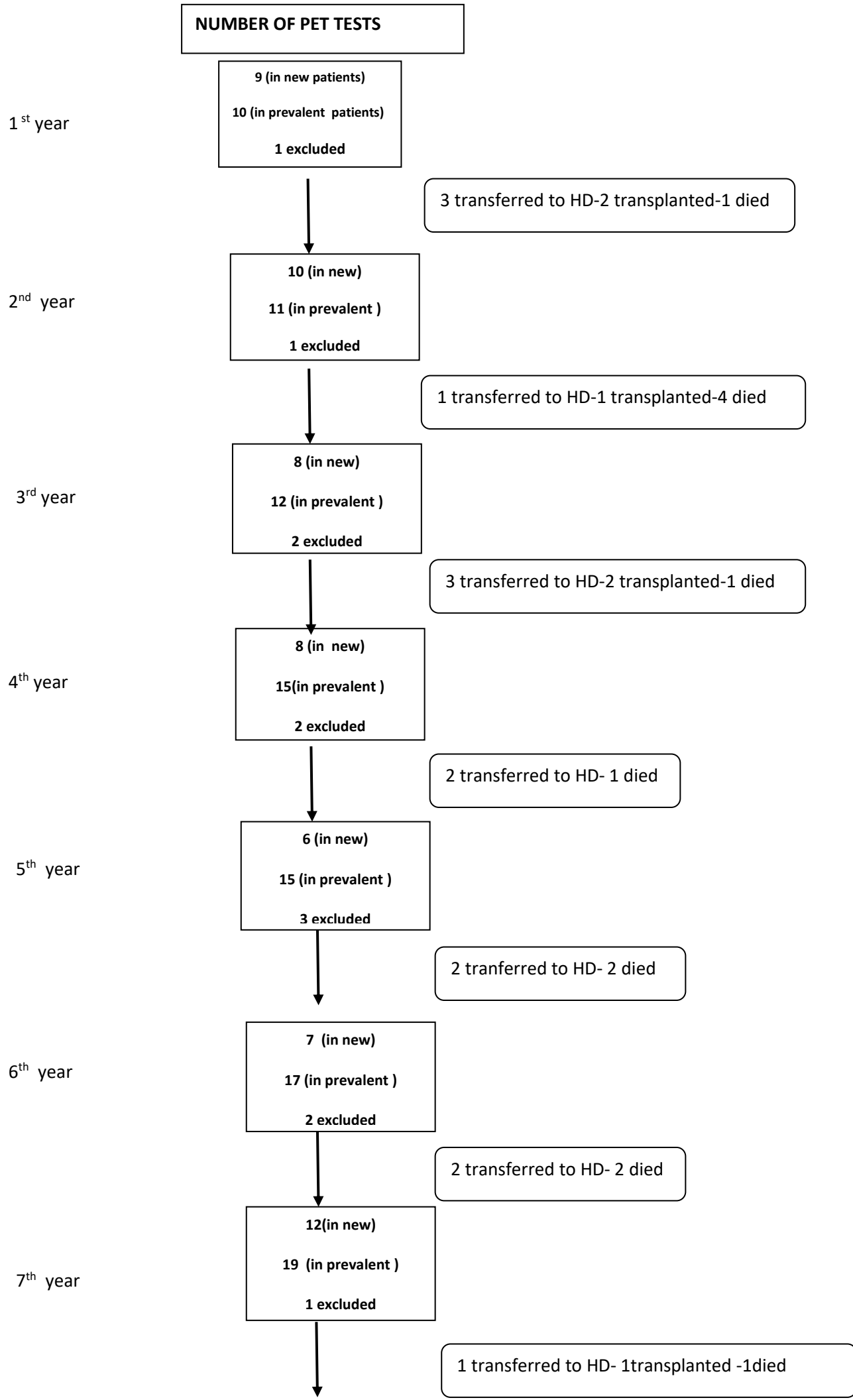

8<sup>th</sup> year

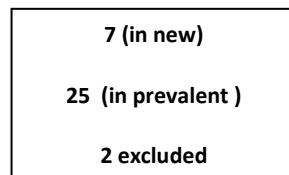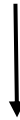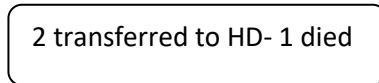

9<sup>th</sup> year

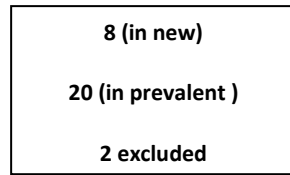

**Figure S1:** Flowchart of patients and PET tests during the study.

Supplement: Supplementary file 1 [file jcm-10-03650-s001.zip › jcm-1330984-supplementary.pdf]
